# Indications, complications, and clinical outcomes of fixation and acute total hip arthroplasty for the treatment of acetabular fractures: A systematic review

**DOI:** 10.1007/s00590-023-03701-z

**Published:** 2023-08-28

**Authors:** Fortunato Giustra, Giorgio Cacciola, Francesco Pirato, Francesco Bosco, Ivan De Martino, Luigi Sabatini, Giuseppe Rovere, Lawrence Camarda, Alessandro Massè

**Affiliations:** 1 Department of Orthopaedics and Traumatology, University of Turin, CTO Torino, Via Zuretti, 29, 10126 Turin, Italy; 2 Department of Orthopaedics and Traumatology, Ospedale San Giovanni Bosco di Torino - ASL Città di Torino, Turin, Italy; 3 Istituto Ortopedico del Mezzogiorno d’Italia “Franco Scalabrino”, Via Consolare Pompea, 98100 Messina, Italy; 4 Ortopedia Protesica e Robotica - Humanitas Gradenigo, Turin, Italy; 5 Department of Orthopaedics and Traumatology, Fondazione Policlinico Universitario A. Gemelli IRCCS-Università Cattolica del Sacro Cuore, Rome, Italy; 6 Department of Orthopaedics and Traumatology (DiChirOnS), University of Palermo, Palermo, Italy

**Keywords:** Combined hip procedure, CHP, Hip surgery, Arthroplasty, Acetabular fracture, THA, Acute total hip arthroplasty, Open reduction internal fixation

## Abstract

**Purpose:**

Acetabular fracture fixation can be challenging, especially in the elderly. Open reduction and internal fixation (ORIF) alone may not allow for early weight bearing and is associated with a high rate of secondary osteoarthritis; therefore, a combined hip procedure (CHP) or ORIF with acute total hip arthroplasty, may be beneficial in this population. The objective of this study was to perform a systematic review of all reported cases of CHP.

**Methods:**

PubMed, Embase, Scopus, and Cochrane databases were searched for studies analyzing acetabular fractures in the elderly managed with a combined hip procedure (CHP). The research was performed following the PRISMA guidelines. The included studies' methodological quality was evaluated using the MINORS score. The present study was registered on PROSPERO.

**Results:**

Eleven clinical studies were included in the final analysis. The mean age was 74.4 (63.2–78) years. Low-energy trauma was the most common mechanism of injury (64%). The most prevalent fracture pattern was the anterior column and posterior hemitransverse (ACPHT) (30.6%). The Kocher-Langenbeck approach was preferred for ORIF of posterior fractures and hip arthroplasty. The ilioinguinal approach and modified Stoppa were generally used for anterior fractures. The overall complication rate was 12.2%, and hip dislocation was the most frequent cause of reoperation (4.4%). The average Harris Hip Score reported postoperatively was 81.6 points, which was considered “good.”

**Conclusions:**

CHP is a safe treatment for elderly acetabular fractures with an acceptable complication and reoperation rate that results in good clinical outcomes.

**Level of evidence:**

Level of evidence IV.

## Introduction

Open reduction and internal fixation (ORIF) of acetabular fractures is challenging, especially in elderly patients with complex fractures and poor bone quality [1–3]. Unfavorable prognostic factors for ORIF in this patient population include articular impaction, comminution, preexisting osteoarthritis, severe osteopenia, or osteoporosis [4].

The management of acetabular fractures in the elderly population depends on the fracture pattern, fracture severity, and the patients’ general conditions [3, 4]. These fractures are typically treated with ORIF if no unfavorable prognostic factors are present, with the goal of preserving the native joint [5, 6]. In cases with one or more negative prognostic factors, a combined hip procedure (CHP), or ORIF with acute total hip arthroplasty (THA), can be considered due to the high risk of secondary osteoarthritis [3–7].

The most appropriate treatment for patients with these injuries is still under debate; ORIF has been shown to have better clinical results, while THA allows for immediate weight bearing and has lower reoperation rate [6, 7]. The purpose of this study was to provide a comprehensive systematic review of the present literature on CHP, to describe the indications, surgical approaches, and clinical and radiological outcomes of this procedure.

## Materials and methods

### Search criteria

This research was conducted following the Preferred Reporting Items for Systematic Reviews and Meta-Analyses (PRISMA) guidelines. The present study was registered in the PROSPERO database: CRD42022385186 [8, 9]. A comprehensive review of PubMed/Medline, Cochrane, Scopus, and Embase databases was performed using the following key terms in association with the Boolean operators AND, OR until December 2022: “acetabular fracture,” “total hip arthroplasty,” “open reduction internal fixation,” “ORIF,” “combined hip procedure,” “CHP,” and “THA.”

### Inclusion and exclusion criteria

Articles that evaluated the clinical outcomes of patients treated with a combined hip procedure (ORIF and THA) for treating displaced acetabular fractures in patients older than 60 years were considered. Inclusion criteria were original articles written in English, which included at least ten patients, and a minimum follow-up of one year. Exclusion criteria were different surgical techniques, such as ORIF or THA alone, unavailable full-text articles, case reports, letters to the editor, biomechanics reports, and instructional course lectures.

### Study screening

**Fig. 1:**
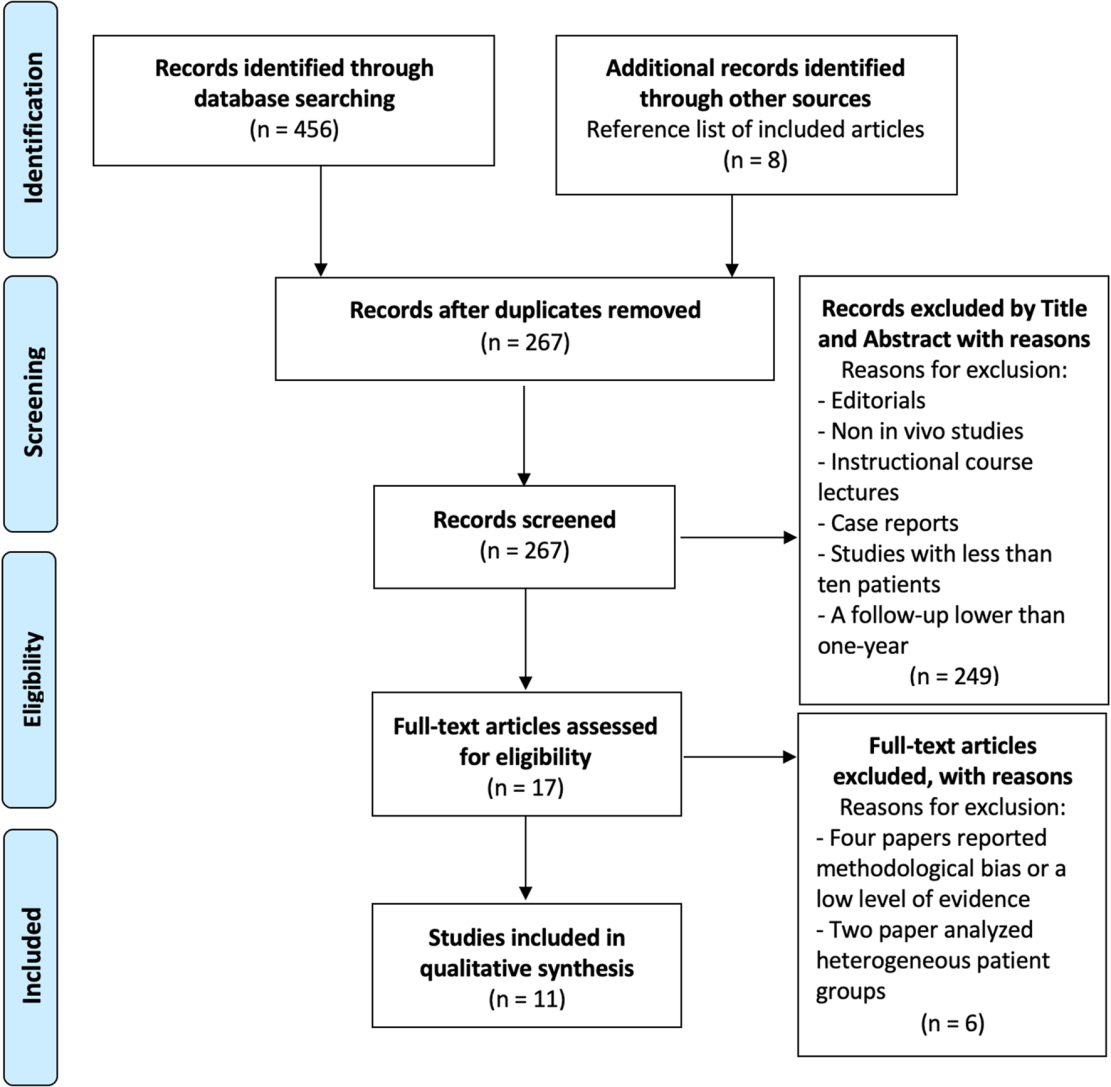
PRISMA flowchart

Two independent authors (GC and FP) independently searched by title and abstract. The full text was obtained and examined if the articles met the inclusion criteria following the title and abstract screening. If the title and abstract of each study did not contain sufficient information to determine its eligibility for inclusion, the full manuscript was examined. A cross-search of the selected articles was also conducted to obtain other articles relevant to the study. In case of disagreement between the two authors, a third author (AA) was consulted, and a consensus was reached. The search yielded 456 studies that were screened to determine the outcome of elderly patients treated with a CHP following acetabular fractures. After eliminating duplicate articles and applying inclusion and exclusion criteria, 11 clinical studies met the inclusion criteria and were included in the final analysis [10–20] (Fig. 1).

### Data collection

For each study, the following data were collected: title, first author, year of publication, level of evidence (LoE), injury mechanism, classification according to Letournel and Judet [21], number of patients, patients who died and were lost to follow-up, age of patients, length of follow-up, indication, surgical approaches and techniques, complications, reinterventions, mortality rate, and patient-reported outcome measures scores (PROMs).

### Methodological quality assessment

The Methodological Index for Non-Randomized Studies (MINORS) score routinely applied in the arthroplasty-related literature [22–24] was used to assess the quality of the included studies. For each question (8 items), the scale assigned a score of 0 if the item is not provided, 1 if the item is partially described, or 2 if the item is well-described. Study quality was assessed by the sum of all items: poor (0–5 points), moderate (6–10 points), and good (11–16 points). The risk of bias using the MINORS score was performed separately by two different authors (GC and FP), and in case of discrepancies, a third author (FB) was consulted.

### Statistical analysis

A descriptive statistical analysis was conducted with R software, version 4.0.5 (2020; R Core Team, Vienna, Austria). Categorical variables were presented as an absolute number and frequency distribution. Continuous variables were presented as means and standard deviation (SD), with the range between minimum and maximum values. A *p*-value < 0.05 was considered statistically significant.

## Results

### Quality assessment

The quality of the eleven included studies [10–20] was assessed, and the mean score was 11.2 (range, 9–12), of which eight were rated good quality. The remaining three studies were rated as moderate quality. No study was classified as poor (Table 1).

**Table 1:** Study characteristics

Author and publication year	LoE	MINORS score	N° of hips, initial cohort/final cohort	N° of hips lost to follow-up and/or died	Age	Gender female	Follow-up
			N°/N°	N°	y.o., Mean ± SD/(range)*	N° (%)	Months, mean ± SD/(range)*
Manson 2022 [10]	III	11	25/22	3	70.7 ± 8.7	8 (36%)	Minimum 12
Hislop 2022 [11]	IV	12	57/57	0	77 (60–95)	24 (42.1%)	Minimum 12
Smakaj 2022 [12]	IV	12	21/21	0	73.4 ± 1.84	16 (76.2%)	Minimum 24
Selvaratnam 2021 [13]	IV	12	14/14	0	63.2 (43–94)	11 (78.6%)	58 (36–132)
Lannes 2020 [14]	III	12	26/26	0	78 ± 6	11 (42%)	12 (1–96)
Borg 2019 [15]	III	12	13/13	0	76.5 (64–89)	5 (38.5%)	Minimum 24
Lont 2019 [16]	IV	12	34/34	0	71 (52–92)	10 (29.4%)	15 (1–82)
Chakracarty 2014 [17]	IV	10	19/19	0	77 (55–92)	6 (31.6%)	22 (2–80)
Rickman 2014 [18]	IV	9	48/24	24	77 (55–90)	8 (33%)	24 (8–38)
Herscovici 2010 [19]	IV	10	24/22	2	75.3 (60–95)	10 (45.5%)	29.4 (13–67)
Boraiah 2009 [20]	IV	11	21/18	3	71 (60–95)	8 (44.4%)	46.8 (12–120)
Overall		11.2	302/270	32	74.5 (53.2–77)	117 (38.8%)	23.8 (12–58)

*LoE* level of evidence, *MINORS* the methodological index for non-randomized studies, *N°* Number, *y.o* years old, *SD* standard deviation

% percentage, * if SD was not reported, values range were recorded

### Demographic and clinical data

A total of 302 hips were initially included. After excluding thirty-two hips (10.6%) due to missing data or patients lost to follow-up, 270 hips were left for analysis. The mean age at the time of surgery was 74.4 (range 63.2–78) years. There were 117 women (38.7%) and 185 men (61.3%). The mean follow-up was 23.7 (range 12–58) months (Table 1). Ten of the eleven included studies reported the mechanism of injury (222 hips) [10–19]. Low-energy trauma from ground-level falls was the most common mechanism in 64% of cases (142 hips), while high-energy trauma was responsible for 36% of cases (80 hips). Ten of the eleven included studies reported fracture classification according to Judet and Letournel’s criteria (245 hips) [11–21]. Associated pattern fractures were more common than simple fractures, with 180 cases (73.5%) and 65 cases (26.5%), respectively. The most common fracture pattern was an anterior column and posterior hemitransverse (ACPHT, 75 cases, 30.6%), followed by both column fractures (39 cases, 15.9%). For simple pattern fractures, posterior wall fractures were the most common (29 cases, 11.8%) (Table 2).

**Table 2:** Fractures classification according to Judet and Letournel classification

Author and publication year	Simple fractures					Associated fractures				
	Anterior wall	Anterior column	Posterior wall	Posterior column	Transverse fracture	T-Type fracture	Both column	ACPHT	Transverse + Posterior wall	Posterior wall + posterior column
Manson 2022 [10]	Not reported									
Hislop 2022 [11]	0	0	0	5	1	0	5	38	0	5
Smakaj 2022 [12]	0	1	2	1	2	2	2	7	1	3
Selvaratnam 2021 [13]	0	0	8	0	0	0	0	0	0	6
Lannes 2020 [14]	0	1	3	0	5	3	5	6	2	1
Borg 2019 [15]	0	0	1	0	0	0	5	5	1	1
Lont 2019 [16]	0	2	2	0	1	19	5	3	2	0
Chakracarty 2014 [17]	0	1	0	0	3	1	4	5	4	1
Rickman 2014 [18]	0	4	1	0	8	0	6	3	0	2
Herscovici 2010 [19]	0	0	0	0	0	0	6	7	9	0
Boraiah 2009 [20]	0	0	12	0	1	0	1	1	2	1
Overall *N* (%)	0	9 (3.7%)	29 (11.8%)	6 (2.4%)	21 (8.6%)	25 (10.2%)	39 (15.9%)	75 (30.6%)	21 (8.6%)	20 (8.2%)

*ACPHT* anterior column and posterior hemitransverse

### Indications and surgical approach

All included studies reported data on factors that should be considered preoperatively before performing a CHP because of the high risk of secondary osteoarthritis after ORIF alone [10–20]. Six factors were identified: preexisting hip osteoarthritis [11, 12, 14, 19], presence of acetabular dome impaction [10–12, 14, 16–20], femoral head impaction [10–12, 14, 15, 17–20], intra-articular comminution [10–12, 14, 15, 17], posterior wall involvement alone or in association with other fracture patterns [10, 11, 13, 17], and preexisting osteopenia/osteoporosis [14, 17, 20] (Table 3).

**Table 3:** Preoperative factors associated with negative prognostic factors for ORIF alone

Author and publication year	Preexisting hip osteoarthritis	Acetabular dome impaction	Femoral head impaction	Intra-articular comminution	Fractures that include involvement of posterior wall	Preexisting osteopenia/osteoporosis
Manson 2022 [10]	R	R	R	R	R	NR
Hislop 2022 [11]	NR	R	R	R	R	NR
Smakaj 2022 [12]	R	R	R	R	NR	NR
Selvaratnam 2021 [13]	NR	NR	NR	NR	R	NR
Lannes 2020 [14]	R	R	R	R	NR	R
Borg 2019[15]	NR	NR	R	R	NR	NR
Lont 2019 [16]	NR	R	NR	NR	NR	NR
Chakracarty 2014 [17]	NR	R	R	R	R	R
Rickman 2014 [18]	NR	R	R	NR	NR	NR
Herscovici 2010 [19]	R	R	R	NR	NR	NR
Boraiah 2009 [20]	NR	R	R	NR	NR	R
Overall R/NR	4/11	9/11	9/11	6/11	4/11	3/11

*R* reported data, *NR* not reported data

All included studies [10–20] reported details of the surgical approaches used for CHP (Table 4). A Kocher-Langenbeck (KL) approach in lateral decubitus was generally used for the fixation of posterior fractures. In three studies [15, 16, 19], anterior pattern fracture fixation was conducted through an ilioinguinal approach. Two studies reported anterior fracture fixation by a modified Stoppa approach [11, 14]. Two studies reported the ilioinguinal or Stoppa approach based on fracture patterns [12, 18]. In one study, the fixation of both anterior and posterior fractures was performed by percutaneous technique [17]. In all included studies [10–20], THA was performed with the KL approach.

**Table 4:** Surgical approaches and implant used during combined hip procedure (CHP)

Author and publication year	Posterior approach	Anterior approach	THA approach	Type of cup	Type of Ring/cage	Type of liner	Type of allograft
Manson 2022 [10]	KL	Levine	KL	Multi hole porous cup	Not	N/A	N/A
Hislop 2022 [11]	KL	Modified Stoppa	KL	Trabecular metal multihole	Not	DM	Morselized femoral head autograft
Smakaj 2022 [12]	KL	Modified Stoppa + Ilioinguinal	KL	Primary cup in case of adequate bone stock	Burch-Schneider ring in 3 cases	N/A	N/A
Selvaratnam 2021 [13]	KL	N/A	KL	Multihole cup	N/A	N/A	Structural autograft
Lannes 2020 [14]	KL	Modified Stoppa	KL	Cemented cup	Burch-Schneider ring	DM	Not
Borg 2019[15]	KL	Ilioinguinal	KL in case of posterior approach; Lateral direct in case of anterior approach	N/A	Burch-Schneider ring	DM	Morselized femoral head autograft
Lont 2019 [16]	KL	Ilioinguinal	KL	N/A	GAP II Reinforcement ring	N/A	Morselized femoral head autograft
Chakracarty 2014 [17]	Percutaneous supine position	Percutaneous supine position	KL	Uncemented cup with 2–3 screws	N/A	Standard poly liners	N/A
Rickman 2014 [18]	KL	Ilioinguinal or Stoppa	KL	Trabecular metal multihole	N/A	Cemented poly liner (35°–15° inclination and 10°–15° anteversion)	N/A
Herscovici 2010 [19]	KL	Ilioinguinal	KL	N/A	Reinforcement ring in few cases	N/A	Morselized femoral head autograft
Boraiah 2009 [20]	KL	N/A	KL	No info for cup; Large femoral head	N/A	Constrained liner avoided	Morselized femoral head autograft

*THA* total hip arthroplasty, *KL* Kocher-Langenbeck, *N/A* not available, *DM* dual mobility

### Complications, reoperations, and revisions

All included studies [10–20] reported data on postoperative complications. The overall complication, reoperation, and prosthetic revision rates were 12.2% (33 cases), 6.3% (17 cases), and 3.7% (10 cases), respectively. The most frequent complication was hip dislocation (12 cases, 4.4%), followed by heterotopic ossification (eight cases, 3%) and periprosthetic joint infection (PJI) (five cases, 1.9%) (Table 5).

**Table 5:** Complication, reoperation, and revision rate after CHP for acetabular fractures

Author and publication year	N° of hips	Overall complications	Overall reoperations	Overall revisions	Dislocation	PJI	Heterotopic ossification	Wound dehiscence	Aseptic loosening	Periprosthetic fracture	Peroneal nerve palsy
		N° (%)	N° (%)	N° (%)	Surgical/Non-surgical	Surgical/Non-surgical	Surgical/Non-surgical	Surgical/Non-surgical	Surgical/Non-surgical	Surgical/Non-surgical	Surgical/Non-surgical
Manson 2022 [10]	22	1 (4.5%)	1 (4.5%)	0 (0%)	0/0	0/0	0/0	1/0	0/0	0/0	0/0
Hislop 2022 [11]	57	8 (q4%)	4 (7%)	2 (3.5%)	1/4	3/0	0/0	0/0	0/0	0/0	0/0
Smakaj 2022 [12]	21	0 (0%)	0 (0%)	0 (0%)	0/0	0/0	0/0	0/0	0/0	0/0	0/0
Selvaratnam 2021 [13]	14	0 (0%)	0 (0%)	0 (0%)	0/0	0/0	0/0	0/0	0/0	0/0	0/0
Lannes 2020 [14]	26	4 (15.4%)	2 (7.7%)	2 (7.7%)	0/2	2/0	0/0	0/0	0/0	0/0	0/0
Borg f2019[15]	13	0 (0%)	0 (0%)	0 (0%)	0/0	0/0	0/0	0/0	0/0	0/0	0/0
Lont 2019 [16]	34	2 (5.9%)	2 (5.9%)	0 (0%)	1/0	0/0	0/0	0/0	0/0	1/0	0/0
Chakracarty 2014 [17]	19	7 (36.8%)	2 (10.5%)	1 (5.3%)	1/0	0/0	0/4	1/0	0/0	0/0	0/1
Rickman 2014 [18]	24	1 (4.2%)	1 (4.2%)	0 (0%)	0/0	0/0	0/0	1/0	0/0	0/0	0/0
Herscovici 2010 [19]	22	8 (36.4%)	4 (18.2%)	4 (18.2%)	2/0	0/0	0/4	0/0	2/0	0/0	0/0
Boraiah 2009 [20]	18	2 (11.1%)	1 (5.6%)	1 (5.6%)	1/0	0/0	0/0	0/1	0/0	0/0	0/0
Overall	270	33 (12.2%)	17 (6.3%)	10 (3.7%)	12 (4.4%)	5 (1.9%)	8 (3%)	4 (1.5%)	2 (0.7%)	1 (0.4%)	1 (0.4%)

*PJI* periprosthetic joint infection, *N°* number, % percentage

### Clinical scores

Eight included studies [10–12, 14–16, 19, 20] reported at least one clinical score. Five studies [10, 12, 14, 19, 20] reported the mean postoperative Harris Hip Score (HHS) (mean 81.6, range 72–92.5). Three studies [10, 12, 15] reported the mean postoperative Short Form-36 score, the mean value for physical component synthesis (PCS) was 48.9 (range 41.3–67.5), while the mean value for mental component synthesis (MCS) was 36.4 (range 12.5–45.6). Two studies [11, 16] reported the mean value of the Oxford Hip Score (OHS) (mean 38.7, range 37.3–41). Two studies [12, 15] reported the mean Pelvic Discomfort Index (PDI) (mean 46.7, range 28.5–57.9). One study reported the mean Western Ontario and McMaster Universities Arthritis Index (WOMAC) (mean 16.5 ± 13.9) [10] (Table 6).

**Table 6:** Patient-reported outcome measure score (PROMs) after combined hip procedure (CHP) for the treatment of acetabular fractures in elderly patients

Author and publication year	N° of hips	HHS	SF-36 PCS	SF-36 MCS	OHS	WOMAC	PDI
	N°	Mean ± SD/(range)*	Mean ± SD/(range)*	Mean ± SD/(range)*	Mean ± SD/(range)*	Mean ± SD/(range)*	Mean ± SD/(range)*
Manson 2022 [10]	22	92.5 ± 7.5	45.3 ± 8.9	45.6 ± 12.9	N/A	16.5 ± 13.9	N/A
Hislop 2022 [11]	57	N/A	N/A	N/A	37.3 (28–48)	N/A	N/A
Smakaj 2022 [12]	21	83.8 ± 2.4	41.3 ± 0.86	41.5 ± 0.41	N/A	N/A	57.9 ± 1.34
Selvaratnam 2021 [13]	14	N/A	N/A	N/A	N/A	N/A	N/A
Lannes 2020 [14]	26	72.4 ± 11.6	N/A	N/A	N/A	N/A	N/A
Borg 2019 [15]	13	N/A	67.5 (42.6–80.7)	12.5 (7.2–62.9)	N/A	N/A	28.5 (17.5–46.5)
Lont 2019 [16]	34	N/A	N/A	N/A	41 (33–46)	N/A	N/A
Chakracarty 2014 [17]	19	N/A	N/A	N/A	N/A	N/A	N/A
Rickman 2014 [18]	24	N/A	N/A	N/A	N/A	N/A	N/A
Herscovici 2010 [19]	22	72 (42–86)	N/A	N/A	N/A	N/A	N/A
Boraiah 2009 [20]	18	88 (78–99)	N/A	N/A	N/A	N/A	N/A
Overll	270	81.6 (72–92.5)	48.9 (41.3–67.5)	36.4 (12.5–45.6)	38.7 (37.3–41)	16.5 ± 13.9	46.7 (28.5–57.9)

*HHS* Harris hip score, *SF-36 MCS* Short Form–36 mental component summary, *SF-36 PCS* Short Form–36 physical component summary, *OHS* Oxford hip score, *WOMAC* Western Ontario and McMaster Universities Arthritis Index, *PDI* pelvic discomfort index, *N°* number, *SD* standard deviation, *N/A* not available

*If SD was not reported, values range were recorded

## Discussion

### Results summary

In this systematic review and meta-analysis, the CHP achieved good clinical results with acceptable complication rates. Acetabular fractures treated with CHP were typically caused by low-energy trauma (64% of cases) and patients had an average age of 74.5 years. The complication, reoperation, and prosthetic revision rates were 12.2%, 6.3%, and 3.7%, respectively. Hip dislocation was the most frequent postoperative complication (4.4%), requiring revision in 50% of cases. The average HHS reported postoperatively was 81.6 points, which was considered good.

### Indications

Open reduction and internal fixation is the standard treatment for displaced acetabular fractures. However, in elderly patients with fractures that have negative prognostic factors, such as articular impaction and preexisting arthritis, the high rate of secondary hip osteoarthritis limits the success of this procedure [25, 26]. There are now a substantial number of studies evaluating [10–20] CHP in this setting warranting this systematic review. We identified six different factors that are used to identify patients the CHP may be appropriate for, including preexisting hip osteoarthritis, acetabular dome impaction, femoral head impaction, intra-articular comminution, fractures that include involvement of posterior wall, and preexisting osteopenia/osteoporosis [27].

Secondary osteoarthritis is the leading cause of reoperation after ORIF for acetabular fractures [28–30]. In a series of ORIF alone, Smakaj et al. [12] reported that 29.2% of patients developed secondary osteoarthritis and required THA. Similarly, Manson et al. [10] reported that 16% of patients treated by ORIF developed secondary osteoarthritis. In a recent systematic review, McCormick et al. [31] reported that patients managed with ORIF, independent from fracture patterns, showed a THA conversion rate of 15% at an average of 16.7 months.

A poor acetabular reduction (> 3 mm gap) can lead to early degeneration of the hip joint and a rapid development of secondary osteoarthritis [32–34]. In young patients with good bone quality, performing an ORIF to obtain an anatomic reduction is essential and not debated [32, 33]. But in case of complex fractures (acetabular dome impaction, femoral head impaction, and intra-articular comminution) in elderly patients with poor bone quality, the CHP may be a superior treatment [34]. Fractures involving the posterior wall, alone or in association with other patterns, such as transverse plus posterior wall, T-Type, ACPHT, and both columns, are associated with a higher risk of non-anatomic reductions and secondary osteoarthritis [34]. Several biomechanics studies reported that even a minimum posterior wall defect significantly increases the superior contact forces leading to early degenerative changes [32–34].

### Surgical approaches

The primary surgical objective is to restore the relationship between the ischium and anterior inferior iliac spine to ensure the stability of the acetabular component. The choice between the anterior and KL approaches depends on the fracture's location and associated comminution. KL is the gold standard approach for managing posterior column area fractures. KL approach allows the direct visualization of the entire posterior column, posterior wall, and supra-acetabular region. Isolated posterior wall, isolated posterior column, associated posterior wall and column, and fractures with posterior wall/column fragment could be treated through the KL approach [35, 36].

On the other hand, the limitations of a KL approach become apparent when dealing with isolated anterior wall/column fractures or when confronting associated fractures accompanied by anterior fragments. The intricacies of such fractures demand alternative strategies beyond the scope of a KL approach [35, 36]. In the case of fracture involving the anterior column fragment, experts like Manson and Chen et al. [37, 38] propose a more comprehensive solution involving reduction and fixation through an anterior approach. Subsequently, the avenue for a successful THA through a KL approach opens up. This hybrid approach capitalizes on the strengths of both techniques to ensure optimal outcomes. In situations where obtaining adequate column stability poses a challenge via the posterior approach alone, the necessity of an anterior approach for ORIF becomes evident. To bolster column stability, recourse to an anterior approach, such as the ilioinguinal approach or the modified Stoppa approach, might become inevitable for addressing anterior wall fractures [10, 12, 37, 38]. This multifaceted approach underscores the adaptable nature of fracture management, tailored to the unique demands of each case.

### Complications

The complexity of the surgery during CHP leads to various and frequent intraoperative and postoperative complications, and outcomes following CHP for acetabular fractures have previously been quite unpredictable. However, all studies and reviews considered postoperative instability and dislocation the most frequent complications and leading causes of revision [39]. The all-cause reoperation-free survivorship was 93.7%, and the all-cause revision-free survivorship was 96.3% at an average follow-up of 23.7 months. We reported a pooled CHP survivorship similar to the revision rate reported in a recent systematic review on acute THA for acetabular fracture (21 studies, 430 acetabular fractures with a revision rate of 4.3% [40]). The reoperation rate of CHP reported in our study is significantly lower than that of ORIF alone, as reported by McCormick et al. [31] who reviewed 19 studies with 1413 patients, and found a 15% rate of conversion to THA.

In our review, recurrent dislocation as a complication was reported in 12 (4.4%) cases, and 50% of them required component revisions. Different authors described many techniques to reduce the risk of dislocation [41, 42]. Three of the eleven studies suggest using dual mobility liners to reduce the risk of recurrent dislocation [11, 14, 15]. Despite using dual mobility liners, Hislop et al. [11] reported a dislocation rate of 23.8% (five cases). Only one required revision of the component due to recurrent dislocation, while the remaining four were managed by closed reduction showing no further dislocation.

### PROMs

Despite the complexity of the surgical procedure and the relatively high complication associated with a CHP for the treatment of acetabular fracture in the elderly, patients showed good function scores. The average HHS, reported by five studies [10, 12, 14, 19, 20], was considered “good.” Manson reported an “optimal” average postoperative HHS with 92.5 points. Two studies reported a “good” average postoperative [12, 20], while two studies reported an average “fair” postoperative HHS [14, 19]. Comparing the average postoperative HHS reported by using CHP with the average HHS reported by McCormick et al. [31] for ORIF alone and THA, there were no significant differences.

Two studies reported the average postoperative pelvic discomfort index (PDI) score. PDI is a 14-items questionnaire developed in 2015 by Borg et al. [15] to evaluate the outcomes following pelvic ring or acetabular fracture. It ranges from 0% (no discomfort) to 100% (maximum discomfort). Manson et al. [10] reported an average PDI score of 57.9 points after two years after surgery, meaning that patients reported “severe discomfort.” On the other hand, Borg et al. [15] reported "moderate" pelvic discomfort, with an average score of 35 points. In both studies, the average PDI was higher, meaning that CHP causes more pelvic discomfort than ORIF alone, but the difference does not reach a statistically significant value [10, 15].

### Limitations

The included research quality is inextricably linked to the quality of this systematic review. A critical flaw in our systematic review was the absence of Level I or II comparative clinical trial studies. In general, selection bias is more likely to occur in Level III and IV research. These limitations are evidenced in the low average MINORS score and methodological issues such as the lack of consecutively examined patients and prospective study designs.

We were unable to do a meta-analysis because there were insufficient homogenous comparison papers available. Additionally, we could not account for several variables that affected the results of our investigation. Some studies differed according to patient characteristics, prostheses, implantation by various surgeons, techniques, and approaches with various rehabilitation programs. Most studies did not explicitly state whether any associated procedures were carried out.

Additionally, our ability to report complications was somewhat constrained by varied (and frequently insufficient) descriptions of complications, radiographic characteristics, and distinctions between minor and major complications. Because the included studies did not describe the results separately by gender or age, no subgroup analysis involving gender or age groups could be carried out.

## Conclusion

In patients with complex acetabular fracture, in the presence of one or more negative prognostic factors for developing secondary osteoarthritis after ORIF alone, CHP provides good hip functionality, quality of life, and an acceptable complication rate. In addition, our data suggest that CHP, in comparison with ORIF alone, reduces the complication and reoperation rates while resulting in similar PROMs. The CHP can be challenging however, and should likely be reserved for tertiary centers where experienced arthroplasty and pelvic trauma surgeons can work together to obtain the most optimal outcomes for these patients.

## Data Availability

The dataset analyzed in this study is available from the corresponding author on reasonable request.
